# Sugar Free: Novel Immunotherapeutic Approaches Targeting Siglecs and Sialic Acids to Enhance Natural Killer Cell Cytotoxicity Against Cancer

**DOI:** 10.3389/fimmu.2019.01047

**Published:** 2019-05-09

**Authors:** John Daly, Mattias Carlsten, Michael O'Dwyer

**Affiliations:** ^1^Department of Hematology, Biomedical Sciences, National University of Ireland Galway, Galway, Ireland; ^2^Department of Medicine, Huddinge, Center for Haematology and Regenerative Medicine, Karolinska Institutet, Stockholm, Sweden

**Keywords:** NK cells, Siglec, sialic acids, immunotherapy, cancer

## Abstract

Natural Killer (NK) cells are cytotoxic lymphocytes that play a key role in the immune system, targeting and destroying invading pathogens and malignantly transformed cells. Evading NK cell-mediated immunosurveillance is therefore critical to facilitating cancer cell survival and metastasis. Signals from a range of inhibitory and activating receptors located on the NK cell surface regulate NK cell cytotoxicity. Recently, attention has turned to the role of hypersialylated tumor cell surfaces in mediating immune-evasion of NK cells. Two inhibitory sialic acid-binding immunoglobulin-like lectin (Siglec) receptors are expressed by NK cells: Siglec-7 and Siglec-9. The abundance of sialic acids on tumor cell surface is hypothesized to regulate NK cell-mediated cytotoxicity by interacting with Siglec-7 and Siglec-9, causing a dampening of NK cell activation pathways. Targeting Siglec-7 and Siglec-9, or the sialic acid coated tumor cell surface is therefore being investigated as a novel therapeutic approach to enhance the NK cell response against cancer. In this review we report on the currently published documentation of the role for Siglec-7 and Siglec-9 receptors on NK cells and their ligands expressed by tumor cells. We also discuss the strategies currently explored to target Siglec-7, Siglec-9 and the sialylated tumor cell surface as well as the impact abrogation of these interactions have on NK cell cytotoxicity against several cancer types.

## Introduction to Natural Killer Cells and Mechanisms Governing Target Killing

**Figure 1:**
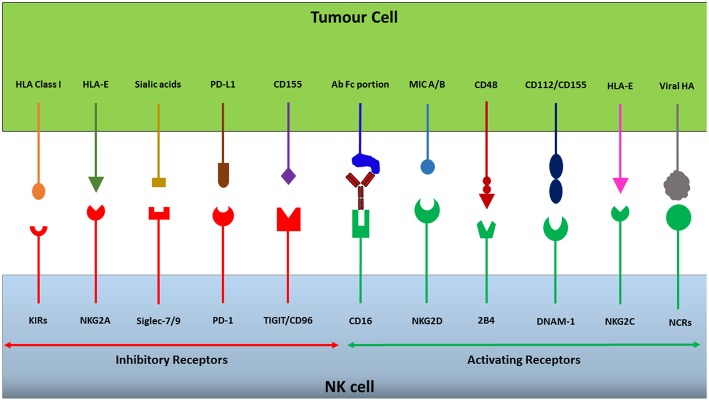
NK cells express a repertoire of activating and inhibitory receptors. The integration of activating and inhibitory signals generated through receptor-ligand interactions dictates whether an NK cell will be triggered to release its cytotoxic granules or not. Cancerous cells can take advantage of this receptor balance to evade immunosurveillance by upregulating or downregulating ligands for specific receptors, changing the overall signal to dampen NK cell cytotoxicity and thereby escape destruction.

Natural Killer (NK) cells are cytotoxic lymphocytes, constituting 5–15% of peripheral blood lymphocytes (1). NK cells play a key role in the immune system, targeting malignantly transformed cells and invading pathogens. Target recognition is dictated by the net sum of signals from an array of inhibitory and activating receptors expressed on the NK cell surface (Figure 1). The wide range of receptors expressed on the NK cell surface and their ligands are reviewed elsewhere (2). If a net signal triggering activation is generated, NK cells release perforin and granzymes from their cytotoxic lysosomes toward the target cell via a process called degranulation (3). Perforin mediates entry of granzymes into the target cell by forming pores in the target cell membrane, and granzymes subsequently cleave intracellular proteins such as caspases thereby activating apoptosis resulting in target cell death. In addition to their role as innate cells being part of the first line defense, activated NK cells also secrete inflammatory cytokines such as tumor necrosis factor alpha (TNF-α) and interferon gamma (IFN-γ) as well as chemokines such as macrophage inflammatory protein-1α (MIP-1α), which are involved in stimulating and recruiting the adaptive immune system (3–6). NK cells can also kill target cells via stimulation of death receptors expressed on the target cell surface. The ligands for these receptors, TNF-related apoptosis-inducing ligand (TRAIL) and Fas-ligand (FasL), are upregulated on the NK cell surface following activation, and induces apoptosis in target cells independently of degranulation (7, 8). The anti-tumor potential of NK cells has since long been established in various pre-clinical models, but their role in clinical medicine has only more recently emerged. The emerging plethora of strategies and drugs, such as the use of cytokines, checkpoint inhibitors, and gene engineering of NK cells (including CAR-NK cells), to bolster endogenous as well as adoptively transferred NK cells will likely be key in further improving the efficacy of NK cell-based cancer immunotherapy.

Normal functioning of the immune system requires an ability to distinguish between “self” and “non-self,” providing protection from threats such as infection and cancer (9, 10). Receptors have evolved that recognize self-associated molecules and self-associated molecular patterns (SAMPs), examples of which are Major Histocompatability Complex (MHC) class I molecules and sialylated glycans, respectively (11). Typically, human NK cells recognize HLA class I molecules (the MHC class I molecules in humans) on target cells as self-associated molecules via a range of inhibitory receptors such as the CD94/NKG2A receptor, inhibitory killer cell immunoglobulin-like receptors (KIRs) and the leukocyte immunoglobulin-like receptor (LIR-1) (Figure 1) (12). The expression of cell surface HLA class I molecules mediates inhibition of autologous NK cells, preventing the destruction of, for instance, healthy somatic cells. However, following malignant transformation, or a viral infection, cells may lose their HLA class I expression (13, 14). The loss of cell surface HLA class I on a tumor cell may lead to avoidance of recognition by cytotoxic T lymphocytes (CTLs), however, at the same time it may render them susceptible to NK cell-mediated immunosurveillance (15). The capacity of NK cells to recognize and eradicate cells lacking HLA class I expression is known as the “missing-self” hypothesis, and is one of the major mechanisms by which NK cells execute immunosurveillance (9).

Based on the originally proposed model for ‘missing-self recognition', in order to kill a target cell NK cells also need activation signals in addition to low or no inhibition during target cell interaction (10). Genetically stressed cells, such as malignantly-transformed cells or virally infected cells, may express and up-regulate the MHC class I chain-related (MIC) ligands MIC-A and MIC-B for the activating natural killer group 2, member D (NKG2D) receptor expressed by NK cells (12). Moreover, other ligands expressed by malignant cells which act to stimulate NK cell activity include CD155 and CD112 which bind to the activating receptor DNAX Accessory Molecule-1 (DNAM-1), with CD155 in particular being overexpressed in both solid and hematological malignancies (16–18), and ligands to the natural cytotoxicity receptors, NKp30, NKp44, and NKp46 that have been shown important for NK cell targeting of several tumor types (18–20). Furthermore, target recognition via antibody-dependent cellular cytotoxicity (ADCC) triggers strong NK cell activation (21). In this situation, the Fc receptor CD16 (FcyRIIIa) expressed by NK cells binds to the Fc portions of antibodies, triggering NK cell secretion of granzymes and perforin to lyse target cells, as well as cytokines to stimulate the adaptive immune response (22).

These examples are just a couple of means by which NK cells interact with a target cell to determine its eventual fate. There are many more inhibitory and activating receptors which have been summarized and discussed elsewhere (2). Nevertheless, it is today clear that the balance between signals from activating and inhibitory receptors on the NK cell surface is crucial for the detection and eradication of malignant cells, while sparing healthy cells.

## The Emerging Role of Siglec Receptors in Regulating NK Cell Cytotoxicity

**Figure 2:**
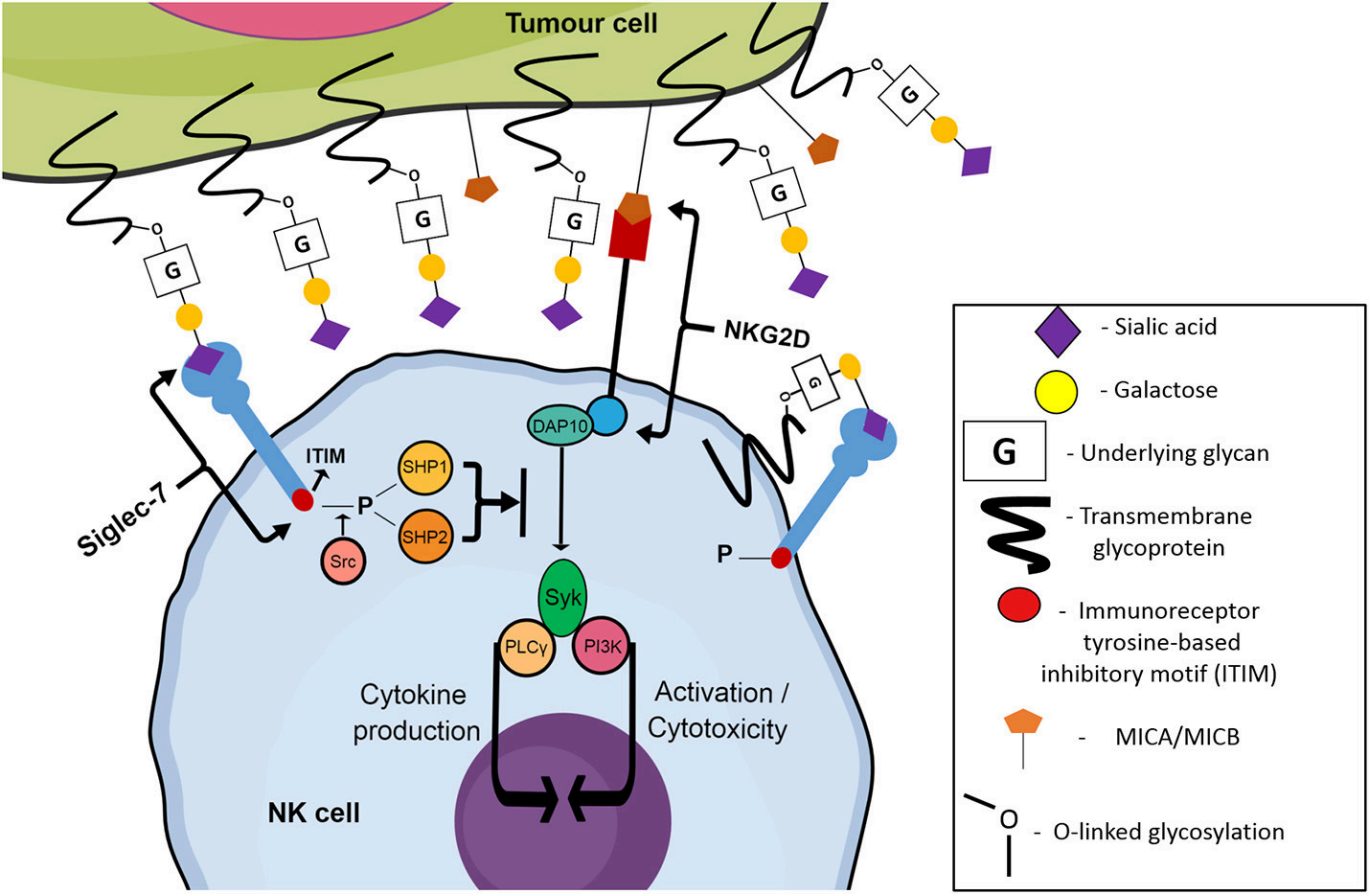
Siglec-7 mediated inhibition of NK cell cytotoxicity towards tumor cells. *Trans* or *cis* binding of Siglec-7 to its cognate ligand results in the Src kinase-mediated phosphorylation of the ITIM motif of Siglec-7. Phosphorylated ITIM sites recruit phosphatases SHP1/2 which inhibit classical NK cell activating pathways such as the NKG2D pathway, stimulated by the binding of NKG2D to stress ligands such as MIC A/B expressed by genetically damaged cells, allowing the tumor cell to escape and continue migrating throughout the circulatory system, eventually reaching new niche sites.

Recently, attention has been drawn to a family of sialic acid-binding immunoglobulin-like lectins, referred to as Siglec receptors (Siglecs). Members of the Immunoglobulin-like protein superfamily capable of binding glycans were originally classified as “I-type lectins.” A sub-type of these lectins specifically recognizing sialic acids were discovered during the mid-to late-1990s, and were classified as Siglecs by Crocker et al. in 1998 (23). Siglecs are a 15-member family of transmembrane receptors, containing both inhibitory and activating members that are found predominantly on hematopoietic cells (24). An N-terminal V-set domain is responsible for the binding of the receptor to sialic acid containing glycans, with a conserved arginine residue facilitating carbohydrate binding activity (23). Apart from resting T cells, most cell types in the human immune system express at least one type of Siglec receptor (23). NK cells express two inhibitory Siglecs: Siglec-7 and Siglec-9, but lack the expression of activating Siglecs (25, 26). Although the specific underlying glycans/proteins which are terminated with sialic acid ligands for both Siglec-7 and Siglec-9 are not fully known, it has been shown that sialic acid moieties on tumor cells have the ability to engage these inhibitory Siglec receptors. Upon binding to cognate ligands, Siglecs trigger the recruitment and activation of intracellular phosphatases via their immunoreceptor tyrosine based inhibitory (ITIM) motif. These phosphatases then act to inhibit NK cell activation pathways, dampening the normally potent cytotoxic ability of the NK cells to target and destroy malignantly transformed cells (Figure 2) (27).

The hypersialylation of membrane-bound glycans and proteins leads to the coating of tumor cells with sialic acid-derived ligands for inhibitory Siglec receptors, resulting in an overall reduction of NK cell activity. This may be especially relevant in the case of tumor cells which have downregulated HLA class I expression, inadvertently heightening their sensitivity to NK cell-mediated immunosurveillance (28). By dampening the NK cell-mediated immune response, malignant cells can traverse the circulation to find new niche sites or evade NK cell recognition in areas of tumor growth, such as the bone marrow (BM), ultimately resulting in the formation of metastases and prolonging cancer cell survival.

Accordingly, targeting Siglecs and modulating hypersialylation have started to generate great interest as potential immunotherapeutic strategies. In this review, the current data relating to the influence of Siglec-7 and Siglec-9 on NK cell-mediated cytotoxicity is summarized, and potential future therapeutic strategies to overcome sialic acid based immune evasion are discussed. Deeper discussions on basic NK cell biology and their role in tumor immunosurveillance and potential in cancer immunotherapy has recently been reviewed elsewhere (28–30) and will therefore only be briefly discussed here.

## Sialic Acids and Hypersialylation in Cancer

Sialic acids are a family of nine-carbon monosaccharides commonly observed terminating glycan chains of glycoproteins and glycolipids on the outer membrane of mammalian cells. Sialic acids are attached to an underlying glycan chain via an enzyme-generated glycosidic linkage (α2-3, α2-6, or α2-8) mediated by a family of over twenty Golgi-located sialyltransferases (31). Given their position and prevalence on the cell's outer surface, sialic acids are thought to act as SAMPs and recognized as markers of cells indigenous to the human body (11). While sialic acids are indeed expressed by normal healthy cells, an abnormally high sialic acid coating on the cell surface is often observed on tumor cells and due to this, hypersialylation of surface-bound glycans and proteins is considered a hallmark of cancer (31, 32).

The importance of hypersialylation in cancer is underlined by the location of the sialylated glycans. Situated on the surface of malignant cells, hypersialylation has been shown to play roles in immune evasion, metastasis and intracellular interactions (31, 32). For example, in addition to mediating NK cell inhibition by interacting with Siglec-7 and/or Siglec-9 receptors, a dense sialic acid coating has also been shown to mask activating NKG2D ligands, preventing the generation of an important activating signal for NK cells (31).

Aberrant sialylation of tumor cells can be mediated by several mechanisms. Overexpression of one of the many sialyltransferases in tumor cells can result in hypersialylation of cell surface glycans. In the case of multiple myeloma (MM), high expression of the sialyltransferase ST3GAL6 has been shown to correlate with poor patient prognosis. Furthermore, successful knockdown of ST3GAL6 severely inhibited homing of MM cells to the BM and ST3GAL6 knockout MM cells showed decreased tumor burden in a murine model (33).

**Figure 3:**
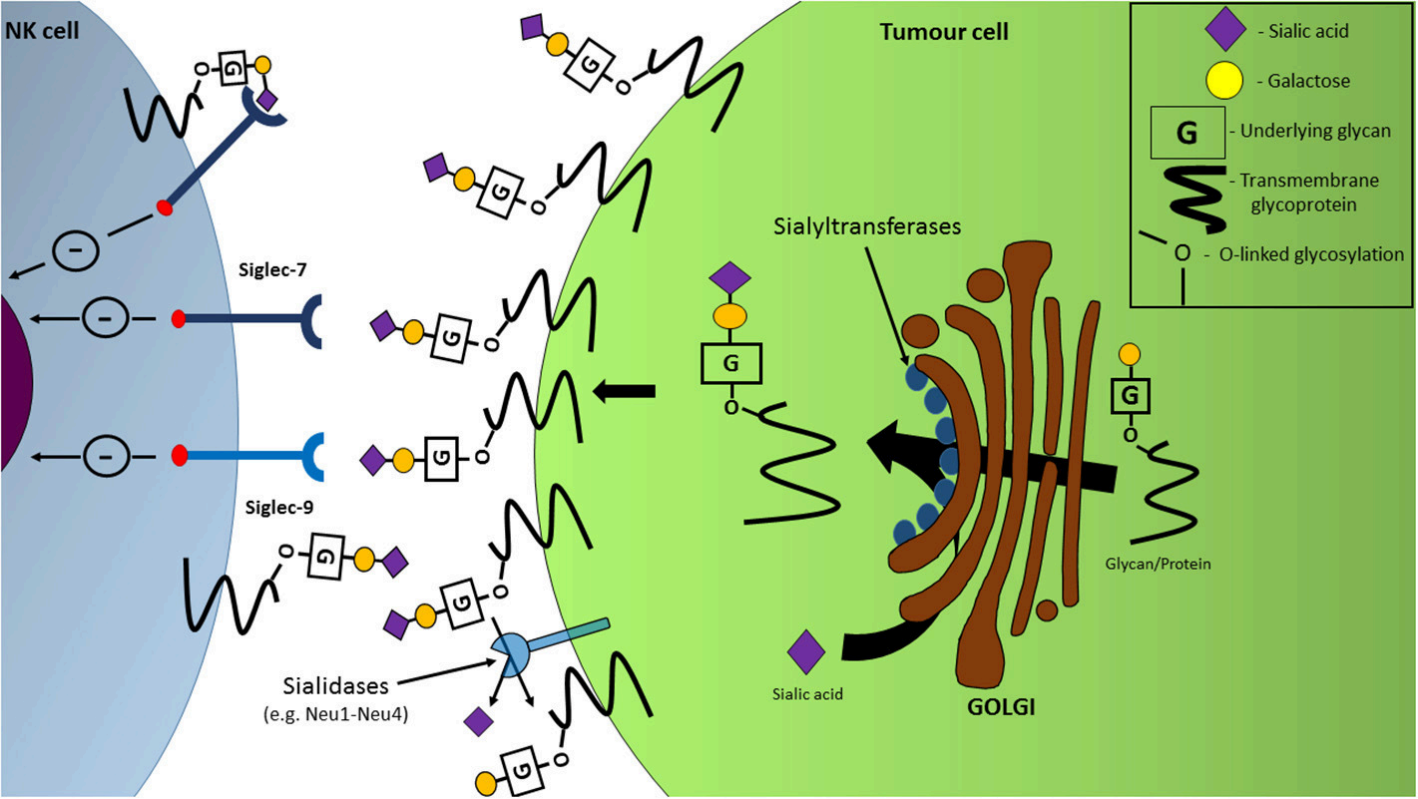
Hypersialylation of tumor cell surfaces facilitates NK cell immune-evasion through interactions with inhibitory Siglec receptors. Upregulation of sialyltransferase expression in the Golgi apparatus, coupled with sialidase dysregulation, leads to an increased expression of sialylated glycans and proteins on the cell surface with the ability to engage inhibitory receptors Siglec-7 and Siglec-9, resulting in a dampening of NK cell functions, including anti-tumor cytotoxicity.

The varying expression levels of sialidases can play a role in the sialylation of tumor cells. Four types of sialidases have been discovered in humans to date: neuraminidases (NEU) 1-4, and function by cleaving sialic acids from glycans via hydrolysis. Of these sialidases, a decrease in the mRNA levels of NEU1 and NEU4 has been recorded in colon cancer cells (34). The decreased expression of sialidases in tumor cells is therefore presented as a means by which a hypersialylated outer surface is generated on malignant cells (Figure 3) (34).

Almarez et al. demonstrated in 2012 that metabolic flux in the sialic acid synthesis pathway, such as increased substrate concentration, can result in increased sialylation of certain protein *N-*glycosylation sites (35). While the latter may not play as important a role in glycan hypersialylation as sialyltransferase/sialidases dysregulation may, it still certainly can be of benefit to tumor cells.

## The Siglec-7 and Siglec-9 Receptors

The Siglec-7 receptor was first described in 1999 as “p75/AIRM1” (AJ007395), a novel 75kDa (“p75”) inhibitory receptor expressed by NK cells belonging to the sialoadhesin family, by Falco et al. (36). Later in 1999, Nicoll et al. first identified Siglec-7 as a member of the Siglec family, based on the similarity of cDNA (AF170485) of the receptor to other Siglec members, and also based on its ability to bind to sialic acids. Nicoll et al. also reported Siglec-7 to be the first Siglec receptor observed on human NK cells (37). Siglec-7 is classified as an inhibitory Siglec due to the presence of an intracellular ITIM motif located on the cytoplasmic tail of the receptor. Activating Siglecs do not express ITIM motifs, but interact with the adaptor protein DAP12 which contains intracellular tyrosine-based activatory motifs (ITAMs), thereby stimulating an activating signal. No activating Siglecs have thus far been observed on NK cells. Since its discovery, Siglec-7 has shown to be expressed on almost 100% of peripheral blood and umbilical cord NK cells, including both the more cytotoxic CD56^dim^ and the more cytokine-producing immune regulatory CD56^bright^ NK cell subsets (38). Although no data has been published on Siglec-7 or Siglec-9 expression on the NK cells of cancer patients, HIV-1 patients have been shown to have significantly reduced levels of Siglec-7 expression (39). However, due to the focus of this review on cancer, this is not discussed in depth here.

The crystallographic structure of Siglec-7 was solved in 2002 by van Aalten et al. (1QFO). Despite being unable to generate a crystal structure of Siglec-7-ligand complex, comparisons between Siglec-7 and sialoadhesin (Siglec-1) allowed for the identification of critical residues needed for ligand specificity (40). The ligand binding site of Siglec-7 lies in the partial opening of the β-sandwich, exposed due to the lack of a stabilizing inter-sheet disulphide. Compared to sialoadhesin, the ligand binding site in Siglec-7 contains more basic amino acid residues—specifically Arg-23, Arg-120, and Lys-135. The additional positivity of the ligand binding site is hypothesized to attract more negatively charged sugars, perhaps explaining the preference of Siglec-7 for disialic acids (40). Arg-124, conserved across all Siglec members, recognizes the carboxyl group of the terminal sialic acid and is a critical residue needed for ligand binding.

The Siglec-9 receptor is another inhibitory Siglec family member, first described in 2000 by Zhang et al. (26). It is structurally similar to Siglec-7, however Siglec-9 also contains one intracellular ITIM motif proximally located near the cell membrane. Another motif, located distally to the membrane, is capable of weakly interacting with SHP1 and SHP2 phosphatases, however the amino acid composition does not fit a typical ITIM motif consensus and thus the motif is not labeled as a classic ITIM (26). Siglec-9 is present on a subset of CD56^dim^ NK cells, whilst minimal expression has been observed on CD56^bright^ NK cells (38). Ligand recognition by Siglecs can be modulated by *cis* interactions with endogenous sialic acid at the cell surface, resulting in the masking of the sialic acid binding site (Figure 2). Indeed, increased levels of Siglec-7 and Siglec-9 have been observed on NK cells of healthy donors treated with the sialidase neuraminidase (38). Being ~80% similar to each other in terms of amino acid sequence, Siglec-7 and Siglec-9 have a similar specificity of binding to both α2,3- and α2,6-linked sialic acids which are attached to glycans by α2,3- and α2,6-sialyltransferases, respectively (26, 41). Siglec-7 has shown a documented preference for internally branched α2,6-linked disialic gangliosides such as the ganglioside DSGb5 (42, 43). However, Siglec-7 also preferentially binds to α2,8-linked disialic acids, with α2,8 sialyltransferases such as ST8Sia1 (GD3 synthase) being responsible for the formation of the α2,8-linkage (40, 44). Whether other α2,8 sialyltransferases are involved in the generation of Siglec ligands is still unclear. The difference in preference, despite the sequence similarity between the two receptors, is accredited to a 6-amino acid stretch located in the C-C' loop of both receptors. The amino acid makeup of C-C' loop is variable between different Siglec members, and van Aalten et al. hypothesize that side chains in the C-C' loop extending toward sub-terminal sugars result in the preference of Siglec-7 and Siglec-9 for differently linked sialic acids (40). The relevant residues thought to be responsible for this in Siglec-7 are Asn-70, Ile-72, and Lys-75 whereas in Siglec-9 the critical residues are Ala-66, Thr-68, and Asp-71 (40).

Sialic acid glycans exposed on the termini of glycosylated glycoproteins, such as mucins (e.g., MUC-1), can function as Siglec ligands. Using recombinant Siglec-7 and Siglec-9 Fc chimeras, ligands for both Siglecs have been shown to be expressed by a range of tumor cell lines, including on renal cell carcinoma (RCC), melanoma, colon adenocarcinoma, cervical cancer, and chronic myeloid leukemia (CML) (38, 43, 45, 46). Of note, as shown for patients with melanoma, Siglec ligand expression was low on both primary melanocytes and peripheral blood mononuclear cells, indicating the cancer restricted nature of expression (38). Ligands for both Siglec-7 and Siglec-9 have also been observed on primary chronic lymphocytic leukemia (CLL) and acute myeloid leukemia (AML) cells (38). Additionally, we have found through analysis of primary samples from both newly diagnosed and relapsed MM patients that Siglec-7 and Siglec-9 ligands are expressed by primary MM cells (47). Having established that inhibition via Siglec receptors engagement can trigger enhanced NK cell immune-evasion, work has been carried out to demonstrate the potential of targeting these inhibitory Siglec receptors as a possible therapeutic strategy to enhance NK cell function against several malignancies (38, 43, 47, 48).

## The Role of the Hypersialylated Cell Surface and Siglec-7 in Regulating the Anti-Cancer Capabilities of NK Cells

De-sialylation of cancerous cells is a good starting point to investigate whether Siglec receptor-ligand interactions may facilitate immune evasion. As Siglec ligands are derived from sialic acids, sialidase treatment (such as with neuraminidase) of cancerous cells is an effective way of stripping sialic acid from glycans and proteins on the outer cell surface. Pre-treatment of cancer cells prior to cytotoxicity assays with NK cells is a common way of investigating the role sialic acids play in evading NK cell-mediated immunosurveillance.

In 2014, Jandus et al. showed that de-sialylation of K562 and HeLa (leukemia and cervical cancer, respectively) cell lines using neuraminidase resulted in a significant increase in healthy donor-derived peripheral blood-NK (PB-NK) cell-mediated cytotoxicity (38). Previous screening of the K562 and HeLa cell lines showed that the cell lines strongly expressed ligands for both Siglec-7 and Siglec-9. Interestingly, there was no increase in PB-NK cell mediated cytotoxicity toward the Siglec-7 and Siglec-9 ligand deficient lymphoblastoid 721.221 cell line upon neuraminidase treatment (38). Furthermore, we have shown that de-sialylation of MM cell lines RPMI8226 and H929 using both neuraminidase and the sialyltransferase inhibitor 3Fax Peracetyl Neu5Ac results in an enhancement of the cytotoxic ability of the NK cell line KHYG-1 against these MM cell lines (47). Prior screening with recombinant Siglec-7 and Siglec-9 Fc chimeras showed the MM cell lines to strongly express ligands for both Siglec-7 and Siglec-9, with KHYG-1 being documented to be partially Siglec-7 positive and Siglec-9 negative. Additionally, blockade of Siglec-7 using anti Siglec-7 Fabs was shown by Jandus et al. to enhance NK-cell mediated cytotoxicity against both K562 and HeLa cell lines, thereby identifying Siglec-7 as a targeted mechanism of NK cell evasion by cancer cells (38).

Another approach to further understand the role for Siglec-7 in regulating NK cell cytotoxicity is to treat the NK cells themselves with a sialidase, thus exposing previously masked Siglec receptors. The sialic acid binding site of Siglec-7 is known to be masked by *cis* interactions (Figure 2) and unmasking has been shown by Kawasaki et al. to reduce the efficiency of NK cells to kill renal cell carcinoma (RCC) cells (43). Neuraminidase-treated NK cells were shown to be less cytotoxic to RCC cell lines strongly expressing DSGb5, compared to RCC cells with low levels of DSGb5 (43). Kawasaki et al. not only showed that Siglec-7 is involved in regulating NK cell activity against RCC but they also identified DSGb5 as a preferred Siglec-7 ligand involved in these interactions.

Similarly, in 2003 Nicoll et al. were able to identify the disialoganglioside GD3 as being involved in NK cell regulation using a murine mastocytoma model in which GD3 was overexpressed. GD3 is found prevalently on cells of the central nervous system but is also highly expressed on melanoma cells (48). Again, Siglec-7 was unmasked using sialidase treatment and when used in cytotoxicity assays against GD3-synthase-transfected P815 murine mastocytoma cells, a Siglec-7 dependent inhibition of NK cell-mediated cytotoxicity was observed (48). Similar to Kawasaki et al. the Nicoll group were also able define a ligand for Siglec-7, whilst showing its relevance in inhibiting NK cell function in another tumor cell line.

Small, high affinity Siglec-7 binding ligands have also been synthesized to inhibit the action of the receptor. Brossmer et al. developed high affinity Siglec-7 ligands and determined that methylsulfonamide was a suitable candidate for biological studies, with high affinity for Siglec-7 and zero toxicity toward either IL-2 activated NK cells or target cells *in vitro* (49). When NK cells pre-treated with the inhibitor were co-cultured in a chromium release assay (a classic assay used to quantify the death of target cells) with the Siglec-7 ligand expressing melanoma cell line Mel1106, increased lysing of target cells was recorded for some, but not all, donors. This was attributed to variable expression of inhibitory receptors between donors and the expression of different Siglec-7 isoforms between donors. Nevertheless, the role of small molecule inhibitors as a means of cancer therapy was shown to be possible. When using the methylsulfonamide the increase in cytotoxicity of NK cells against target cells was lower than reported by groups who have used an anti-Siglec-7 blocking antibody (38, 48). However, Brossmer et al. credited this partially to the antibodies having a much lower *K*_d_ value than methylsulfonamide, and hypothesized that next generation small molecule inhibitors with a lower *K*_d_ value would be able to elicit higher cytotoxic responses from NK cells (49). In the same year Sato et al. also synthesized a small molecule Siglec-7 inhibitor, using disialic acid on polysaccharide dextran (DiSia-Dex). They showed that it was a potent inhibitor of Siglec-7 binding to GD3 and demonstrated that, furthermore, it was able to release Siglec-7 from a Siglec-7-GD3 complex (50). They credited this to a slow dissociation rate and high association rate, but no studies were carried out into the effect DiSia-Dex has on NK cell cytotoxicity against cancer cells. Nevertheless, the potential of small inhibitor molecules in preventing Siglec-7 mediated inhibition of NK cell function has been highlighted and remains a potential future therapeutic strategy.

Collectively, the importance of Siglec-7 in regulating NK cell activity against tumor cell lines has been well-documented in pre-clinical models today. However, its role *in vivo*, especially its impact in facilitating tumor cell immune-evasion, remains to be established. Such studies will likely require the use of xenograft models as murine NK cells do not express Siglec-7 or Siglec-9 (23). Given the recent attention, we believe it is only a matter of time before the role for the Siglec-7 receptor and its ligands *in vivo* is also detailed.

## Siglec-9: Its Role in NK Cell Regulation and Other Immune Domains

To date, several ligands for Siglec-7 have been identified and shown to be expressed on different types of cancers. In contrast, Siglec-9 is known to bind to the mucin MUC16, commonly expressed on the surface of epithelial cells such as tracheal and ovarian cells (51). Cell surface MUC16 (csMUC16) is expressed by ovarian cancer cells and is detectable in the peripheral blood of cancer patients. Indeed, a peptide found in the VNTR domain on both csMUC16 and shedded MUC16 (sMUC16) is considered the tumor marker and is known as cancer antigen 125 (CA125) (51). CA125 is known to be heavily glycosylated with approximately a quarter of its overall mass estimated to constitute carbohydrates, in particular *O*-linked glycans although *N-*linked glycans were also observed on CA125 isolated from ovarian cancer cells (52). Belisle et al. showed in 2010 that neuraminidase-treated sMUC16 binding to peripheral blood mononuclear cells derived from ovarian cancer patients was reduced compared to heat-inactivated neuraminidase, leading them to hypothesize that the cognate receptor for MUC16 bound to sialic acids and eventually identifying Siglec-9 as a receptor for MUC16 (51). Additionally, by conducting mass spectrometry on Siglec-9 ligands isolated from carcinoma cell lines, Läubli et al. identified the heavily-glycosylated protein Lectin Galactoside-binding soluble 3-binding protein (LGALS3BP) as a novel ligand for Siglec-9 (46). LGALS3BP was also found to bind to recombinant Siglec-5 and Siglec-10, however this is beyond the scope of this review as neither are expressed by NK cells.

The role that Siglec-9 plays in regulation of NK cell cytotoxicity is hence not as well-understood as for Siglec-7. This may be in part because of the partial expression of Siglec-9 on circulating peripheral blood NK cells, of which only 40–50% of CD56^dim^ NK cells are positive for this receptor (38). Nevertheless, independent and specific Fab antibody blockade of Siglec-9 by Jandus et al., has demonstrated that blockade of Siglec-9 was sufficient to strongly enhance NK cell cytotoxicity against the K562 and HeLa cell lines (38).

Overall, this is the totality of what is understood today of the role Siglec-9 plays in regulating NK cell activity against cancerous cells. Targeting Siglec-7 may represent a more attractive therapeutic strategy due to its near-ubiquitous expression on NK cells, while Siglec-9 is only expressed on a subset of NK cells. However, due to the similarity of the two inhibitory receptors—with over 80% of their amino acid chains being identical it is conceivable that the same therapeutic strategy developed for Siglec-7 could be simply modified as to target both Siglec-7 and Siglec-9, enhancing the cytotoxic capabilities of NK cells in a stronger fashion than targeting a single Siglec would offer.

Despite less evidence supporting Siglec-9's role in facilitating NK cell immune-evasion by cancer cells currently existing than for Siglec-7, Siglec-9 potentially modifies other immune cell types, resulting in the creation of a more favorable environment for tumor formation. For example, MUC1 carrying the sialylated core 1 glycan (MUC1-ST) on breast cancer cells, was recently shown to bind to Siglec-9 on primary human monocytes and macrophages, inducing a secretome signature unique to each cell type. Moreover, when MUC1-ST bound to Siglec-9 expressed by primary macrophages, an M2 tumor associated macrophage phenotype was actively induced, as shown by the inhibition of CD8^+^ T-cell proliferation and upregulation of the expression of indoleamine 2,3-dioxygenase (IDO), the scavenger receptor CD163, the mannose receptor CD206 and the immunological checkpoint ligand PD-L1 (53). Hence, more research is needed to improve our understanding of these two Siglec receptors on NK cells *per se*, and the role of the Siglec family in the immune system in general. Increased understanding of the impact Siglecs have in regulating the immune response is crucial to fully exploit the therapeutic possibilities this biology would offer.

## Novel Therapeutic Strategies to Overcome Sialic Acid-Based Evasion of NK Cells

As discussed previously, de-sialylation of several cancerous cell lines enhances their susceptibility to NK cell tumor immunosurveillance. A novel therapeutic strategy to enhance the functions of NK cells could simply be to specifically target and de-sialylate cancerous cells. Siglec-7, and to an extent Siglec-9, appear to play roles in regulating NK cell activity, however, simply targeting tumor cells for de-sialylation may represent an easier method to counter the inhibitory effects imposed by hypersialylation.

For example, in 2016 Bertozzi et al. conjugated a sialidase (Sia) isolated from *Vibrio cholerae* with the human epidermal growth factor receptor 2 (HER2) targeting therapeutic monoclonal antibody Trastuzumab (Tras). In several HER2^+^ breast cancer cell lines the Tras-Sia complex was observed to successfully abolish ligands for both Siglec-7 and Siglec-9, as well as enhancing NKG2D binding (54). Additionally, the Tras-Sia complex was able to augment NK cell antibody-dependent cell mediated cytotoxicity toward breast cancer cells compared to Tras alone, further potentiating their cytotoxicity by preventing inhibitory Siglec receptor/ligand interactions as well as stimulating ADCC (54). Targeted delivery of antibody-sialidase conjugates to cancerous cells is a promising approach to enhance NK cell activity, with the dual-purpose function of stripping inhibitory Siglec ligands as well as presenting antibody Fc chains to stimulate NK cell-mediated ADCC.

The utilization of nanoparticles as a therapeutic delivery strategy has also been investigated as a means of combatting hypersialylation. Targeted delivery of the sialyltransferase inhibitor 3Fax-Peracetyl Neu5Ac to melanoma cells resulted in an ~75% decrease in cancerous nodes per lung of mice in a murine model, in comparison to PBS treated controls (55). The nanoparticle design allowed slow release of 3Fax-Peracetyl Neu5Ac in the *in vivo* model, highlighting that a sustained inhibition of sialyltransferases is possible dependent on nanoparticle design. Nanoparticle delivery also reduces the risk of off-target toxicity, principally nephrotoxicity, which appears to be the main dose limiting toxicity of systemic treatment with 3Fax-Peracetyl Neu5Ac (56). In a more recent study by Büll et al., using the same 3Fax-Peracetyl Neu5Ac inhibitor to treat C57BL/6JRccHsd WT mice transfected with a melanoma cell line, sialic acid blockade via intra-tumoral injection resulted in supressed melanoma tumor growth and enhanced tumor cell killing by CD8^+^ T-cells (57). In addition, sialic acid blockade resulted in increased numbers of tumor infiltrating-NK cells and CD8^+^ T-cells with a decrease in regulatory T-cells also observed (57). The enhanced infiltration of immune cells into the tumor upon de-sialylation highlighted here demonstrates the immunosuppressive effects of hypersialylated cancer cell surfaces and the realistic possibility of targeting sialic acid expression becoming a novel means to target cancer cells.

Of note, none of the studies published to date have presented any data indicating a potential side effect of blocking Siglec receptor/ligand interactions. Furthermore, no evidence supporting that blockade of this interaction detunes the function of NK cells has been proposed and is it therefore likely an attractive pathway to interfere with in contrast to inhibitory KIRs. Future data will hopefully shed light on these aspects of developing viable therapeutic approaches to unleash NK cells against tumor cells via blockade of the inhibitory Siglec-7 and Siglec-9 receptors *in vivo*.

## Conclusion

There is now compelling evidence that the hypersialylated glycan layer on cancer cells plays an important role in facilitating immune evasion. The discovery and recent focus on targeting inhibitory Siglec members has identified these receptors as potentially important new immune checkpoints, amenable to therapeutic targeting in cancer. Work published in this area thus far indicates that this approach may be relevant to a wide range of cancers. However, full documentation of Siglec ligands and their expression on cancer cells is needed to increase our understanding of the inhibitory pathway and to help advance the development of viable therapeutic approaches. Targeted strategies to eliminate Siglec ligand expression on cancer cells merit further research and clinical development, while evaluation of checkpoint inhibition strategies as well as the adoptive transfer of NK cells engineered to eliminate Siglecs could prove an effective way of improving current NK cell-based approaches for the treatment of cancer.

## Author Contributions

JD, MC, and MOD conceived the idea, carried out the research, and wrote the manuscript. MC and MOD reviewed and approved the manuscript.

### Conflict of Interest Statement

MOD is a director and owns equity in Onkimmune. The remaining authors declare that the research was conducted in the absence of any commercial or financial relationships that could be construed as a potential conflict of interest.
